# Levels of plasma ADAM10, education, and cognitive performance of healthy older adults and persons living with Alzheimer’s disease from a middle‐income country

**DOI:** 10.1002/alz.092667

**Published:** 2025-01-09

**Authors:** Marcia Regina Cominetti, Renata Valle Pedroso, Pedro Henrique Moreira Victoriano, Danilo Barroso de Sousa, Leticia Fernanda Palma, Marina Mantellatto Grigoli, Cecilia Patricia Popolin, Vanessa Alexandre da Silva, Sabrina Dorta de Oliveira, Patricia Regina Manzine, Lucas Nogueira de Carvalho Pelegrini

**Affiliations:** ^1^ Federal University of São Carlos, São Carlos Brazil; ^2^ The Global Brain Health Institute, Trinity College Dublin, Dublin, Dublin Ireland; ^3^ Federal University of São Carlos, São Carlos, SP Brazil; ^4^ Federal University of Sao Carlos, Sao Carlos, SP Brazil

## Abstract

**Background:**

Blood‐based biomarkers are gaining attention lately, and recent evidence supports the potential role of ADAM10 as an Alzheimer’s disease (AD) biomarker. However, most available information on these biomarkers comes from high‐income countries, where the population is usually highly educated. The influence of years of education (YoE) and older age on cognitive performance is well described in the literature, and they are risk factors for dementia. Therefore, in this study, we aim (1) to analyze whether education mediates the relationship between age and cognitive performance even among those with few YoE and (2) to compare plasma levels of ADAM10 in healthy controls (HC) and persons living with AD (PLwAD).

**Methods:**

In this cross‐sectional and analytical study, participants (n=81) were low‐educated older adults from a middle‐income country (Brazil) and were assigned into two groups: HC (n=37) and PLwAD (n=44). The Mini‐mental State Examination (MMSE) evaluated cognitive performance. SDS‐Page and Western‐Blotting were used to obtain plasma levels of ADAM10.

**Results:**

Participants’ mean age and YoE were 75.5 (7.9) and 4.9 (3.5) respectively. PLwAD patients were older (t=‐4.67; p<0.0012), had fewer YoE (t=3.53; p=0.002), performed worse in MMSE (t= 1.72; p<0.001), and had higher levels of ADAM10 in plasma (t=‐2.24; p=0.31). The mediation analysis suggested a total effect of age on MMSE score of ‐0.366 (p<0.001; 95%CI [‐0.53 to ‐0.20]), a direct effect of age (controlling for YoE) on MMSE score of ‐0.302 (p<0.001; 95%CI [‐0.47 to ‐0.13]) and an indirect effect of age on MMSE score, via YoE, of ‐0.0803 (95%BCaCI [‐0.18 to ‐0.01]). Finally, the logistic regression analysis suggested that higher levels of plasma ADAM10 increase the probability of being diagnosed with AD (Exp(B) = 2.099; 95% C.I. for Exp(B) [1019 to 4.321]).

**Conclusion:**

Our results suggested that education mediates the effect of age on cognitive performance, even among those with fewer YoE. Also, higher levels of ADAM10 in plasma are associated with AD diagnosis. These findings hold significance as they offer a distinctive perspective from a middle‐income country with diverse demographics, potentially shaping future research and informing healthcare strategies in analogous settings.

